# Human Bladder Uroepithelial Cells Synergize with Monocytes to Promote IL-10 Synthesis and Other Cytokine Responses to Uropathogenic *Escherichia coli*

**DOI:** 10.1371/journal.pone.0078013

**Published:** 2013-10-14

**Authors:** Benjamin L. Duell, Alison J. Carey, Samantha J. Dando, Mark A. Schembri, Glen C. Ulett

**Affiliations:** 1 School of Medical Science, Griffith University, Gold Coast, Queensland, Australia; 2 Institute for Glycomics, Griffith University, Gold Coast, Queensland, Australia; 3 School of Chemical and Molecular Biosciences, University of Queensland, Brisbane, Queensland, Australia; The University of Melbourne, United States of America

## Abstract

Urinary tract infections are a major source of morbidity for women and the elderly, with Uropathogenic *Escherichia coli* (UPEC) being the most prevalent causative pathogen. Studies in recent years have defined a key anti-inflammatory role for Interleukin-10 (IL-10) in urinary tract infection mediated by UPEC and other uropathogens. We investigated the nature of the IL-10-producing interactions between UPEC and host cells by utilising a novel co-culture model that incorporated lymphocytes, mononuclear and uroepithelial cells in histotypic proportions. This co-culture model demonstrated synergistic IL-10 production effects between monocytes and uroepithelial cells following infection with UPEC. Membrane inserts were used to separate the monocyte and uroepithelial cell types during infection and revealed two synergistic IL-10 production effects based on contact-dependent and soluble interactions. Analysis of a comprehensive set of immunologically relevant biomarkers in monocyte-uroepithelial cell co-cultures highlighted that multiple cytokine, chemokine and signalling factors were also produced in a synergistic or antagonistic fashion. These results demonstrate that IL-10 responses to UPEC occur via multiple interactions between several cells types, implying a complex role for infection-related IL-10 during UTI. Development and application of the co-culture model described in this study is thus useful to define the degree of contact dependency of biomarker production to UPEC, and highlights the relevance of histotypic co-cultures in studying complex host-pathogen interactions.

## Introduction

Urinary tract infections (UTIs) are a major cause of morbidity, affecting 40% of women, of which 20% experience at least one reoccurrence at a later time. Uropathogenic *Escherichia coli* (UPEC) accounts for approximately 80% of all cases of acute UTI such as cystitis and pyelonephritis [1-4], and up to 86% of asymptomatic infections [5]. Acute UTI can be localised to distinct areas of infection, predominantly urethritis and cystitis, with less common but more severe complications sometimes arising from pyelonephritis and urosepsis. Increasing antibiotic resistance of UPEC has highlighted a need for different approaches to combat infection [6,7]. This, in part, has led to investigations of the immunomodulatory properties of cytokines produced during UTI. Several cytokines are intimately involved in the pathogenesis of infection, as reviewed elsewhere [8-11]. The regulatory cytokine, Interleukin-10 (IL-10), which is produced during UPEC infection in murine models of UTI and in patients with UPEC cystitis and pyelonephritis, has been a focus of several recent pathogenesis studies [12-14].

IL-10 regulates immune responses during many infections, predominantly by shifting immune responses towards a Th2-centric adaptive immune outcome that may benefit the host, and sometimes the pathogen [15,16]. IL-10 is produced by a wide variety of leukocytes [16], and can be secreted by multiple intracellular trafficking pathways under different conditions [17]. UPEC induces IL-10 in the bladder during acute UTI, and this has been proposed to down-regulate inflammatory responses shortly after infection via monocytes/macrophages [12], and mast cells [18]. These studies combined *in vivo* analyses of UTI in mice with bladder transcriptomics to identify active biological pathways during infection, which were shown to comprise IL-10 signaling among the top hits in canonical pathway recognition. These studies also used *in vitro* cell culture assays to demonstrate UPEC-triggered up-regulation of IL-10 production in monocytes, but not uroepithelial cells [12].

*In vivo* studies yield valuable insight into disease pathogenesis. However, analyses of cellular interactions between microbes and multiple host cell types that can generate cytokine responses also benefits from *in vitro* methodology. In prior studies of UPEC infection, this has typically used monocultures of uroepithelial cells, monocytes, macrophages, or neutrophils to investigate cytokine responses following infection [19-27]. These studies have offered key insight into cytokine responses to UPEC infection, including how the bacterium utilizes virulence factors such as α-hemolysin to inhibit *in vitro* epithelial cytokine production [28]. Such insight has implications for suppressing the innate immune response during UTI [29,30]. Co-culture of different host cell types in various arrangements, which can rely on stratification and/or co-localisation to generate responses to external stimuli [31-33], offer further advantages for infection studies to mimic *in vivo* responses [12]. Co-ordinated effects of co-localised cells can lead to synergistic interactions between epithelial or endothelial cells and phagocytic or immune cells to drive responses that are not discernable in monocultures [31,32]. 

The current study investigated IL-10 and other cytokine responses in bladder uroepithelial cell and monocyte co-cultures challenged with UPEC. We sought to define the nature of the interactions that occur between these two cell types in response to UPEC, and whether the interactions might comprise additive, antagonistic or synergistic effects in relation to cytokine production. We found that human bladder uroepithelial cells synergize with monocytes in contact-dependent and soluble interactions to promote the production of IL-10 and several other biomarkers based on novel responses that have implications for understanding UTI pathogenesis. 

## Materials and Methods

### Cell lines and Bacteria

The cell lines used in these experiments were the immortalised human lines 5637 bladder carcinoma (ATCC HTB-9), MC116 B-cells (ATCC CRL-1649), Jurkat T-cells (ATCC TIB-152) and U937 monocytes (ATCC CRL-1593.2). All cell lines were purchased from the ATCC (USA), and routinely maintained at low passage number from frozen stocks. For some experiments, U937 monocytes were matured to macrophage-like cells using phorbol-12-myristate-13-acetate in methods as described elsewhere [34]. The UPEC strain used in this study was the prototype CFT073 strain (ATCC 700928), originally isolated from a patient with pyelonephritis [35]. This strain has been used in multiple *in vitro* and *in vivo* UPEC pathogenesis studies [36-39]. Bacteria were routinely grown from frozen glycerol stocks on LB agar and in LB broth at 37°C shaking at 200 rpm.

### Cell culture

To investigate *in vitro* host-pathogen interactions, we routinely used 96-well plate co-cultures in total volumes of 250 μl for infection assays. The routine maintenance and preparation of cells was performed according to methods as described in previous studies [12]. In initial assays, we used a four cell co-culture model (bladder uroepithelial cells, B-cells, T-cells and monocytes) to investigate the contribution of the different cell types to the IL-10 responses to UPEC *in vitro*. In subsequent assays, we removed or substituted individual cell types from this model in various combinations, ensuring that the overall cell numbers and culture media volumes remained consistent between assays, to investigate the contribution of specific cell types to the response. 

For all assays, cells were infected with UPEC using a MOI of 10 (that equated to a total of 1.5 x 10^6^ cfu per well) unless otherwise stated. Cell culture supernatants were collected by centrifugation (500 x *g* 10 min 4°C) at 5 h after infection, and were stored at -80°C until ELISA. We used a commercially available human ELISA kit to quantitate IL-10 protein (88-7106-88 eBioscience). For co-culture assays that were based on the physical separation of uroepithelial cells and monocytes, we used 24-well cell culture plates containing membrane inserts (0.4 μm pore-size 140620 Nunc). In these assays, the co-cultures contained total volumes of 500 μL RPMI1640 per well, and comprised 2 x 10^5^ 5637 cells in the upper insert and/or 1 x 10^5^ U937 cells in the lower well, with infected wells having an MOI of 10 (3 x 10^6^ cfu/mL). Cultures were routinely incubated with UPEC for 5 h at 37°C with 5% CO_2_. Supernatants were then collected and frozen for subsequent ELISA or alternative biomarker analysis. Biological replicates were performed in triplicate (as a minimum) for all assays, and experiments were repeated at least three times. Data presented represent pooled data from multiple independent experiments, as indicated for each figure. 

### qRT-PCR

We used qRT-PCR to analyse IL-10 mRNA gene expression in the different co-cultured cell types *in vitro* following UPEC infection. For these assays, 24-well plate co-cultures were prepared using membrane inserts and infected with UPEC for 5 h as described above. Following infection, the supernatants were collected for biomarker analysis (below), and RNA was prepared from uroepithelial and monocyte populations using a commercial RNA extraction kit (732-6820 Bio-Rad). Total RNA was quantified using a NanoDrop ND-1000 UV-Vis spectrophotometer (NanoDrop Technologies) and 100 ng of RNA from each sample was converted to cDNA using oligo DT primers from a cDNA conversion kit (18080-051 InVitrogen). Subsequent qPCR was performed to quantify the relative abundance of IL-10 mRNA transcripts using the Quantifast SYBR Green PCR kit (204054 Qiagen). The primer sequences used for the amplification of IL-10 and GAPDH, which was used as a housekeeper gene, are described elsewhere [12] and were purchased from Sigma. Reactions were run on a Roche LightCycler 480, using reaction cycling conditions as follows: 95 °C for 5 min followed by 45 cycles of [94 °C for 10 s; 63 °C for 30 s; 72 °C for 20 s] and melt-curve analysis. Relative quantification was performed using ΔCt values in the equation 2.0^-[∆Ct]^ to calculate the relative mRNA expression level of IL-10 in comparison to GAPDH [40-42]. The infection data was expressed as averaged ratios of GAPDH-normalized IL-10 Ct values against the non-infected controls. Quintuplicate biological samples were assayed in technical triplicate. Primer efficiency for GAPDH was 1.86 and 1.85 for IL-10.

### Biomarker analysis

IL-10 levels in initial assays were measured using ELISA, as per the manufacturer’s instructions. In initial assays, we discovered a novel synergistic interaction between bladder uroepithelial cells and monocytes for UPEC-induced IL-10 synthesis. To complement the IL-10 data, we subsequently undertook a quantitative analysis of a comprehensive set of biomarkers to better define the extent of synergistic responses in the infected dual co-culture model. Using supernatants collected from 24-well insert dual co-cultures infected as described above, we analysed a panel of 27 human biomarkers using a BioPlex 27-plex kit (M50-0KCAF0Y BioRad). Biological replicates (n=8) were collected from three independent experiments. These data are presented as individual data points for each biological replicate with a horizontal bar of the mean to illustrate within-group variance.

### Statistics

Welch’s Independent t test was used to analyse mean cytokine levels for ELISA data with significance level set to p < 0.05. Mean cytokine levels (pg/mL) of qRT-PCR and BioPlex data that were not normally distributed were compared using a Mann–Whitney U test. All statistical analyses were carried out using IBM SPSS Statistics software (Version 20.0), and GraphPad Prism software package 5.0. Statistical significance was accepted as p < 0.05. 

## Results

### UPEC-induced IL-10 production in uroepithelial cell co-culture

**Figure 1:**
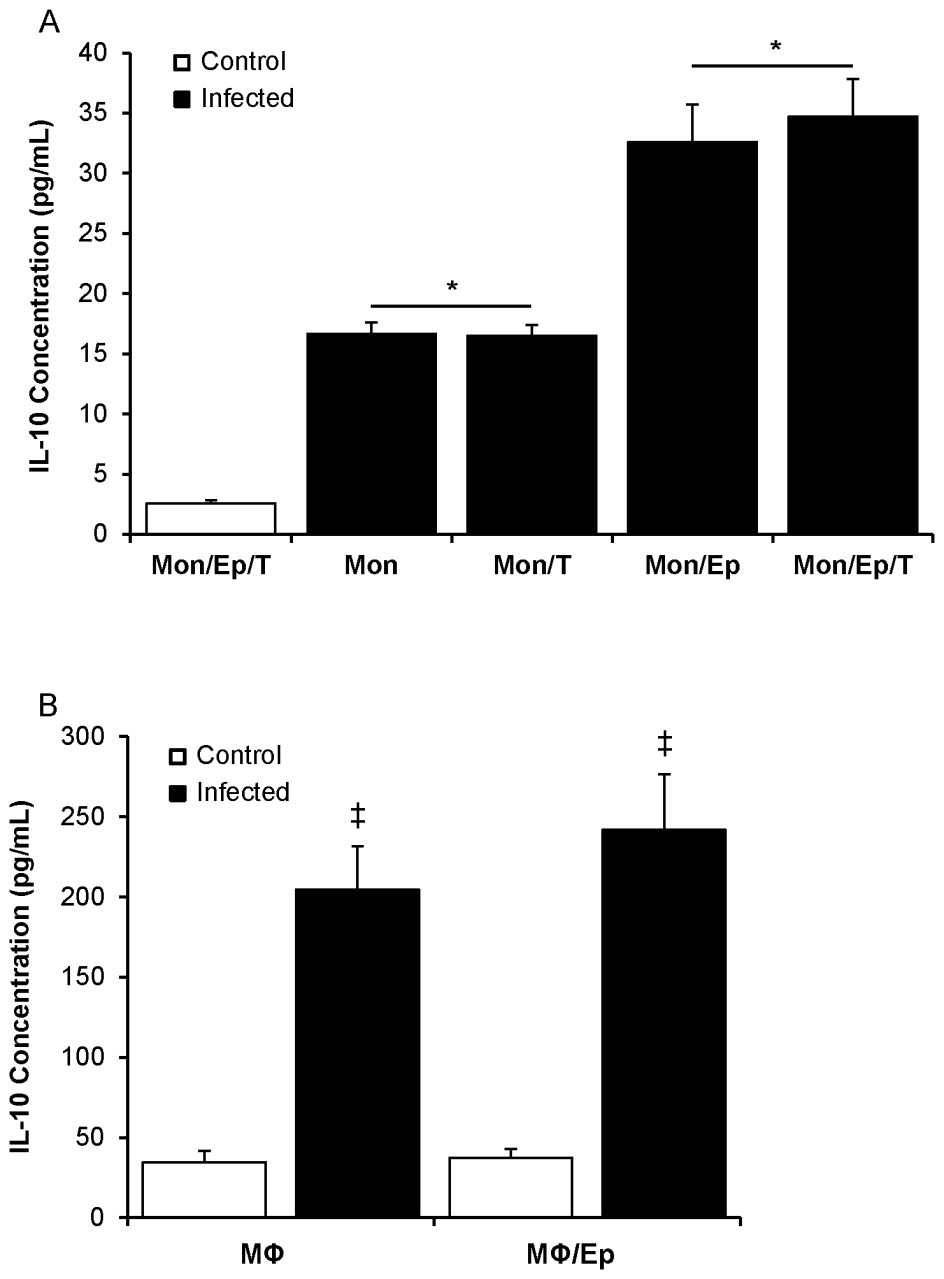
Different cellular combinations reveal synergism between uroepithelial cells and monocytes for UPEC-induced IL-10 synthesis. (A) Uroepithelial cells and monocytes in 96 well microtitre plate co-cultures produced peak levels of IL-10 protein (32-34 pg/mL) compared to monocultures that yielded 16-17 pg/mL following infection. T-cells did not increase IL-10 levels. (B) Substitution of monocytes with macrophages showed much higher levels of IL-10 with or without uroepithelial cells (> 200 pg/mL), but there was no significant difference between the two infected conditions (all infected groups significantly above controls, p ≤ 0.001; monocyte monocultures vs. monocyte co-cultures with uroepithelial cells, * p = 0.002; macrophage monocultures vs macrophage-uroepithelial dual co-cultures, ^‡^ p = 0.003; SEM bars). (Mon = monocyte, Ep = uroepithelial cell, T = T-cell, MΦ = macrophage, Control = uninfected cells).

In a prior study of UPEC-infected uroepithelial cell-monocyte co-cultures, monocytes were the predominant cell type producing IL-10 at the transcriptional level [12]. In the current study, we performed initial comparisons to examine other relevant cell types within a four cell co-culture model comprising uroepithelial cells, monocytes, B- and T-cells and analysed their effects on UPEC-induced IL-10 protein levels in co-culture supernatants. Preliminary assays showed that B-cells secreted basal levels of IL-10, which is consistent with prior data [43]. The inclusion of B-cells in co-cultures caused an approximate doubling of IL-10 levels and the proportional increases were equal in control and UPEC infected conditions (Figure S1A). These data led us to exclude B-cells from subsequent co-cultures. In dual and triple co-cultures of uroepithelial cells, monocytes and T-cells, in which the cells were combined in various groupings using 1.5-1.65 x 10^5^ total cells per well (89:10:1 ratio), we found significant increases in IL-10 in response to UPEC infection, as shown in Figure 1A. In these assays, dual co-cultures of monocytes and T-cells had a total cell number of 1.5-1.65 x 10^4^ cells per well; in monocultures, the cell numbers used were equivalent to those used in co-cultures (i.e. ~10^5^ uroepithelial cells, ~10^4^ monocytes, 10^3^ T-cells). All monocultures and co-cultures containing monocytes produced ≥16 pg/mL IL-10 in response to infection (Figure 1A). This was significantly less than monocyte co-cultures containing uroepithelial cells, which produced 32-34 pg/mL in response to infection (p < 0.001; Figure 1A). The presence of T-cells in these co-cultures did not cause any increase in IL-10 levels. All other co-culture combinations not shown in Figure 1A (excluding those with B-cells) exhibited IL-10 levels below the detection limit of the assay (2 pg/mL) (Controls shown in Figure S1B). Collectively, these data show a previously undescribed synergistic interaction between uroepithelial cells and monocytes for UPEC-induced IL-10 production; i.e. uroepithelial cells in monoculture do not produce IL-10 in response to UPEC infection, however the addition of monocytes to uroepithelial cells doubles the response of monocytes alone to UPEC infection. Based on the synergistic effect between uroepithelial cells and monocytes, we excluded T-cells from subsequent assays. 

### Synergism for IL-10 production in monocyte vs. macrophage uroepithelial co-cultures

Given the heightened response of macrophages for cytokine production in general compared to monocytes [44], we analysed the effect of maturing the monocytes towards IL-10 production in UPEC-infected co-cultures. These data, shown in Figure 1B, showed stronger IL-10 synthesis in macrophage-containing co-cultures compared to monocyte co-cultures following infection (≥ 200 pg/mL vs 16-35 pg/mL; compare Figure 1AB). Importantly, however there was no statistically significant difference between the IL-10 levels in the infected macrophage monocultures and macrophage-uroepithelial cell co-cultures, indicating that the synergistic effect for UPEC-triggered IL-10 is restricted to monocyte-uroepithelial co-cultures; i.e. no significant synergistic phenotype for IL-10 synthesis occurs in macrophage-uroepithelial cell co-cultures.

### Contact-dependency of uroepithelial cell-monocyte synergistic IL-10 responses

**Figure 2:**
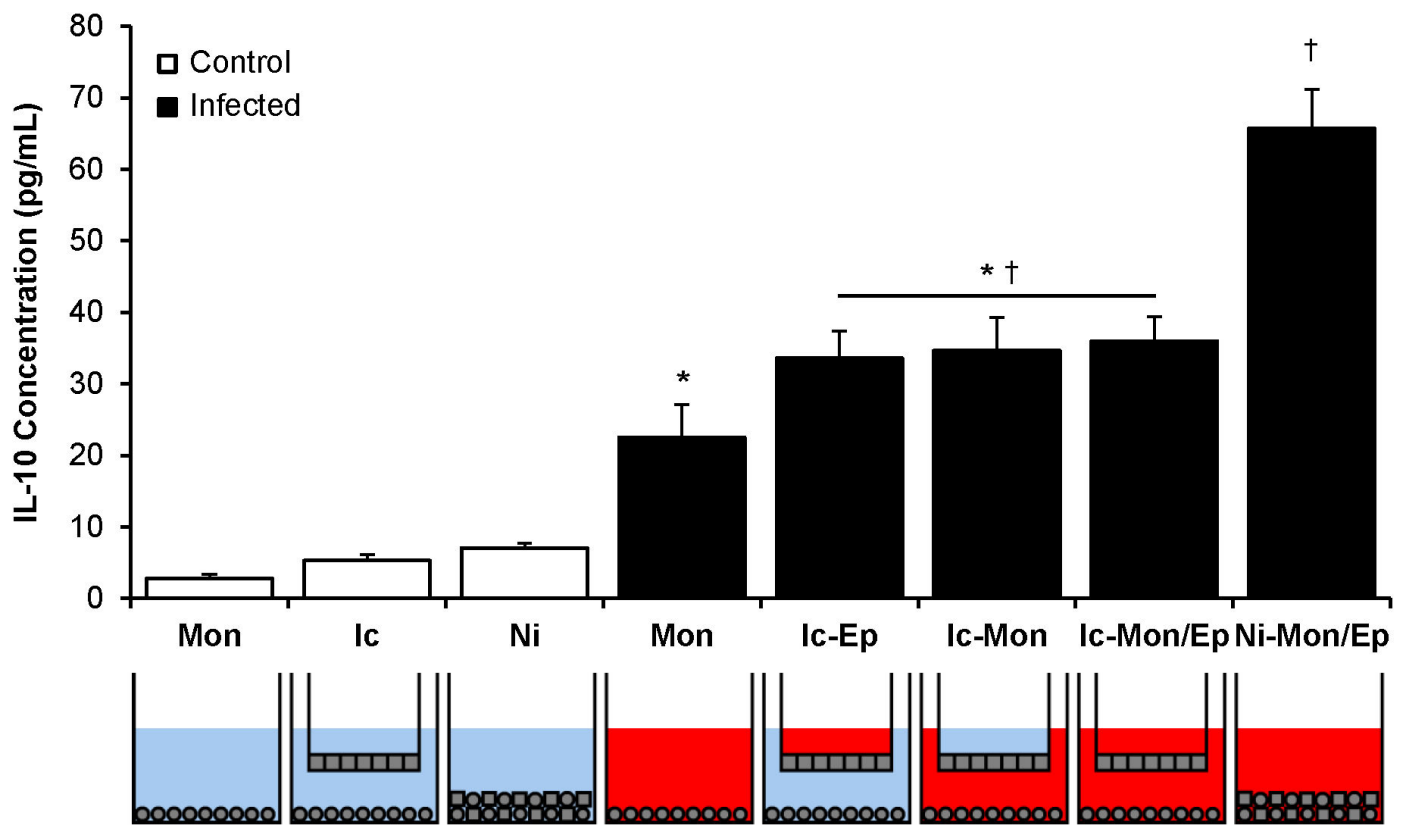
Use of membrane inserts demonstrates contact-dependent IL-10 synergism in UPEC-infected uroepithelial cell-monocyte dual co-cultures. The level of IL-10 produced in co-cultures where uroepithelial cells and monocytes were in contact was double that of cultures where inserts were used to physically separate the two cell types. The schematics below graph show the cell type, infection and presence of inserts; blue = non-infected, red = infected (all control vs infected groups, p < 0.001; monocyte monocultures vs insert-containing dual co-cultures, * p ≤ 0.024; insert-containing dual co-cultures vs no insert dual co-cultures, ^†^ p ≤ 0.002; SEM bars).

In further characterizing the uroepithelial cell-monocyte dual co-culture model, the cell ratios were next adjusted to a 70:30 ratio of uroepithelial cells:monocytes in 96-well plates (rather than 90:10), to mimic inflammatory conditions of infected bladder that are known to encompass a monocyte cellular infiltrate [45] and extensive monocyte transcriptional antibacterial responses [46,47] (and unpublished data). Detailed assays using these cell ratios yielded similar results for IL-10, with a proportional increase in IL-10 responses in infected versus control co-cultures (data not shown). This 70:30 dual co-culture model was then applied to 24-well membrane insert culture conditions to determine whether the synergistic IL-10 response to UPEC infection depended on contact between uroepithelial cells and monocytes. Insert-containing co-cultures routinely comprised uroepithelial cells in the upper compartment, and/or monocytes in the lower compartment, as illustrated in the schematic wells depicted in Figure 2. In these assays, non-infected monocytes produced low levels of IL-10 (2-3 pg/mL at detection limit of the assay), which increased to 20 ± 3 pg/mL in infected monocyte monocultures (Figure 2). Infection of insert-containing wells was performed by adding UPEC to the upper compartment (uroepithelial cells), lower compartment (monocytes), or both. In all of these conditions, IL-10 nearly doubled in production to approximately 35 pg/mL; the levels were similar regardless of which compartment(s) were infected. In infected dual co-cultures without inserts, IL-10 levels increased to 65 pg/mL (Figure 2). Upper compartment infection of uroepithelial cell monocultures resulted in no significant IL-10 levels (Figure S1C). Together, these data show that both contact-dependent and soluble signalling mechanisms between uroepithelial cells and monocytes (i.e. UPEC cannot pass through inserts) promote synergistic induction of IL-10 in response to UPEC.

### IL-10 mRNA expression in UPEC-infected uroepithelial cell monocyte co-cultures

**Figure 3:**
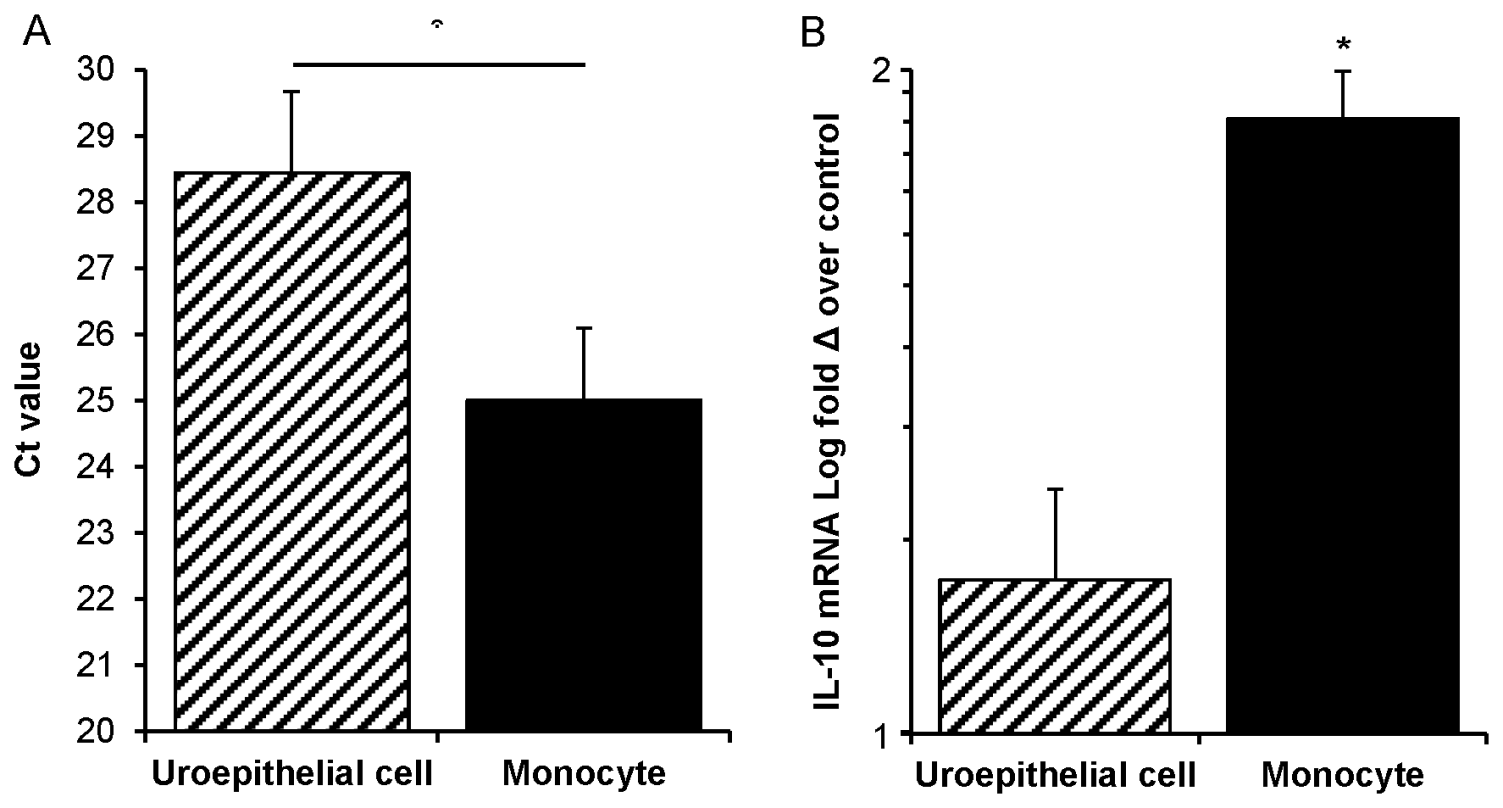
IL-10 mRNA responses to UPEC in dual co-cultures that contain membrane inserts are principally monocyte-derived. (A) Monocytes in dual co-cultures overall have > 10-fold abundance of IL-10 transcript compared to uroepithelial cells, as transcripts emerge 3.4 cycles (Ct = 25.0 vs Ct = 28.4) earlier in qPCR (monocyte vs uroepithelial cell Ct values, p = 0.007). (B) Monocytes from all insert-containing infected conditions showed a statistically significant increase in IL-10 mRNA (1.8-fold increase of averaged infected monocyte conditions over control non-infected monocytes; insert-containing infected monocytes from dual co-cultures vs uninfected control, * p = 0.009). Uroepithelial cells from insert-containing infected conditions did not show significant increases over non-infected (1.16-fold increase of averaged infected uroepithelial cell conditions over control non-infected uroepithelial cells). Mann–Whitney U-tests were used for comparisons and SEM bars are shown.

To determine whether monocytes are the cell type responsible for producing IL-10 in the cell separation model, as was previously reported in non-separated dual co-cultures [12], we undertook a transcriptional analysis. For this, control and UPEC-infected dual co-cultures containing inserts were used for qRT-PCR assay for IL-10 and housekeeper GAPDH gene transcripts. These data demonstrated that monocytes had a significantly higher abundance of IL-10 transcripts compared to uroepithelial cells (Figure 3A; p ≤ 0.001, compare average cycle number of crossover threshold), and produced effectively all of the IL-10 mRNA in this model (Figure 3B). There were significant increases in IL-10 mRNA expression in infected versus non-infected monocytes under conditions where either the upper insert, or lower wells, or both were infected (81-98% increase, equivalent to a 1.8-fold increase on average comparing non-infected to infected monocyte conditions; p ≤ 0.009). In contrast, there were no significant differences in the levels of IL-10 mRNA expression (a 1.16 fold increase on average comparing non-infected to infected uroepithelial cell conditions). Amplification efficiencies of the primers for the different target sequences were within +/- 10% of each other using the assay conditions as described (1.85 vs. 1.86). Thus, monocytes contribute effectively all of the IL-10 transcriptional responses in this co-culture model.

### Biomarker panel analysis of UPEC-induced uroepithelial cell-monocyte synergism

**Figure 4:**
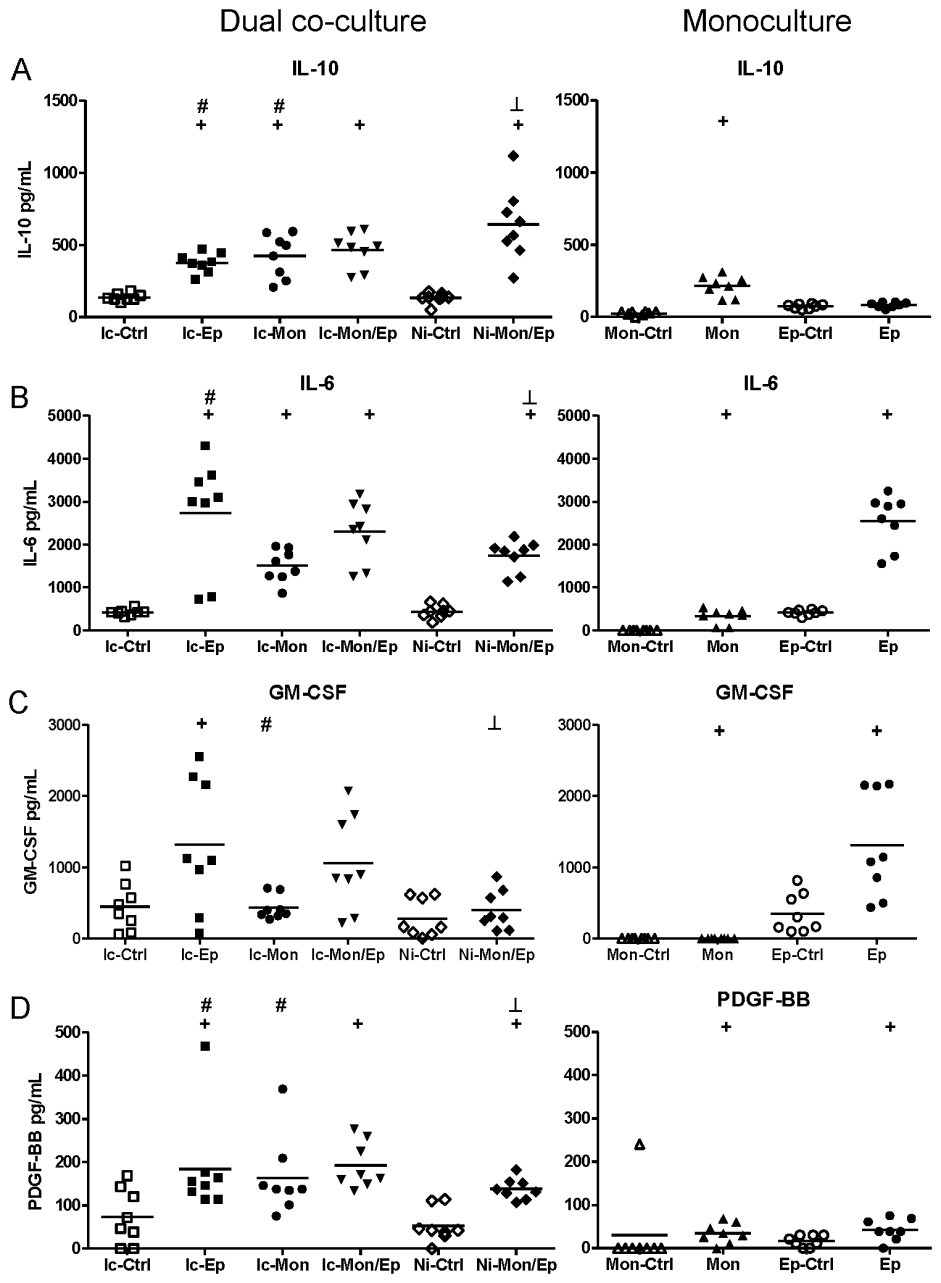
Contact between uroepithelial cells and monocytes drives synergistic and antagonistic biomarker production in UPEC-infected dual co-cultures. (A) IL-10 production was synergistic in co-cultures that showed significant increases mediated by contact between uroepithelial cells and monocytes (compare Ic-Mon/Ep with Ni-Mon/Ep, p ≤ 0.0139). In contrast, the production of IL-6 (B), GM-CSF (C) and PDGF-BB (D) in insert-containing co-cultures was significantly higher than non-insert co-cultures (compare Ic-Mon/Ep with Ni-Mon/Ep), revealing antagonistic effects mediated by contact between monocytes and uroepithelial cells. Statistical comparisons are: ^+^ control vs corresponding infected; ^#^ insert-containing infected co-culture vs corresponding infected monoculture; ^┴^ insert-containing infected co-culture vs no insert infected co-culture, notations are p < 0.05; Mann–Whitney U-test. (Ic-Ctrl = insert-containing dual co-culture uninfected, Ic-Ep = insert-containing uroepithelial infected, Ic-Mon = insert-containing monocyte infected, Ic-Mon/Ep = insert-containing monocyte and uroepithelial infected, Ni-Ctrl = no insert co-culture uninfected, Ni-Mon/Ep = no insert monocyte and uroepithelial infected, Mon-Ctrl = monocyte uninfected, Mon = monocyte infected, Ep-Ctrl = uroepithelial uninfected, Ep = uroepithelial infected).

**Figure 5:**
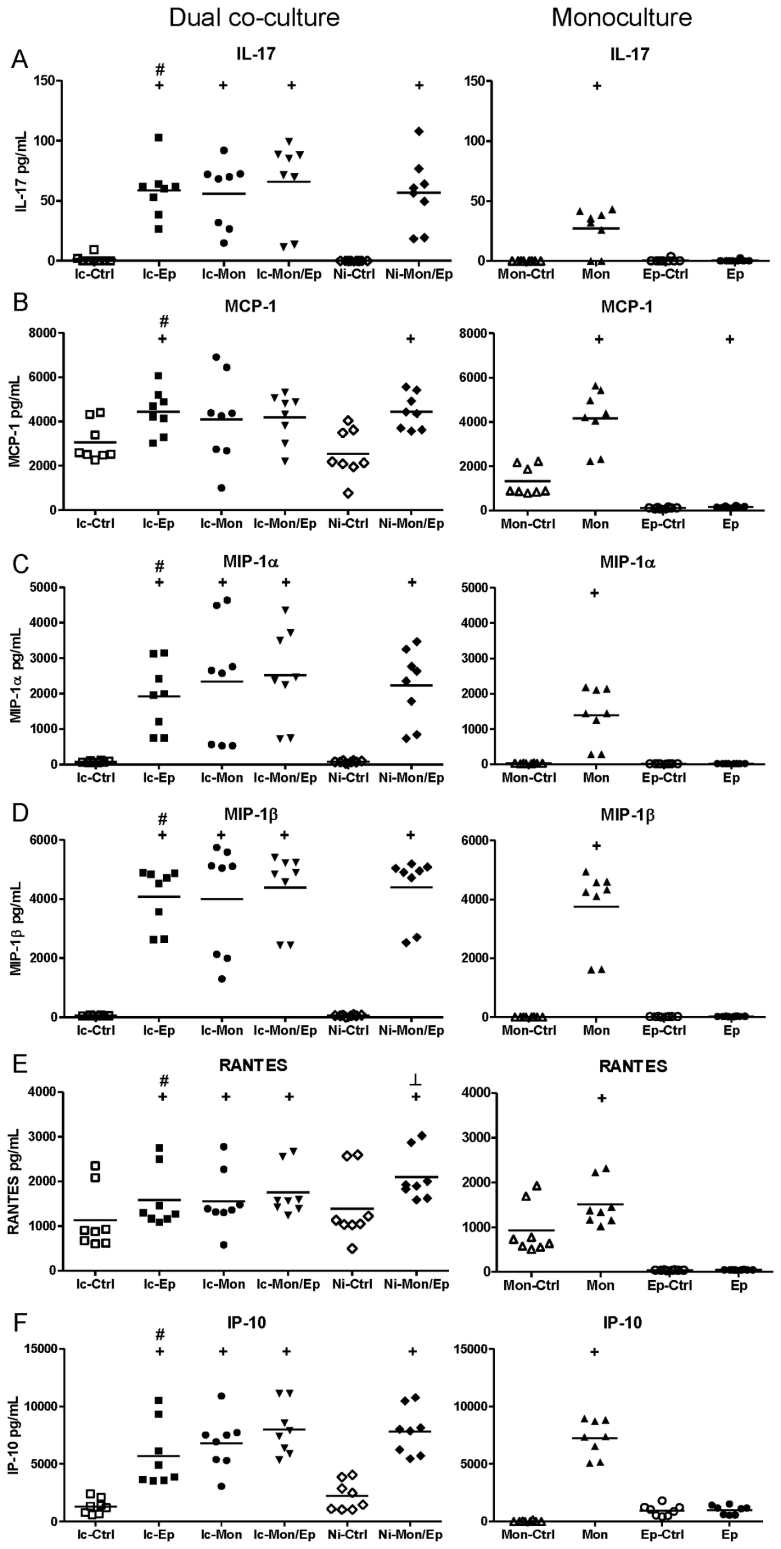
UPEC-induced soluble-dependent biomarker responses in dual co-cultures grouped according to biomarker levels in uroepithelial monocultures. Experimental Details are as per Figure 4. In this dataset, the most common type of response was a synergistic increase based on a soluble factor(s) conferred by presence of uroepithelial cells. Statistical comparisons are: ^+^ control vs corresponding infected; ^#^ insert-containing infected co-culture vs corresponding infected monoculture; ^┴^ insert-containing infected co-culture vs no insert infected co-culture, notations are p < 0.05; Mann–Whitney U-test.

**Figure 6:**
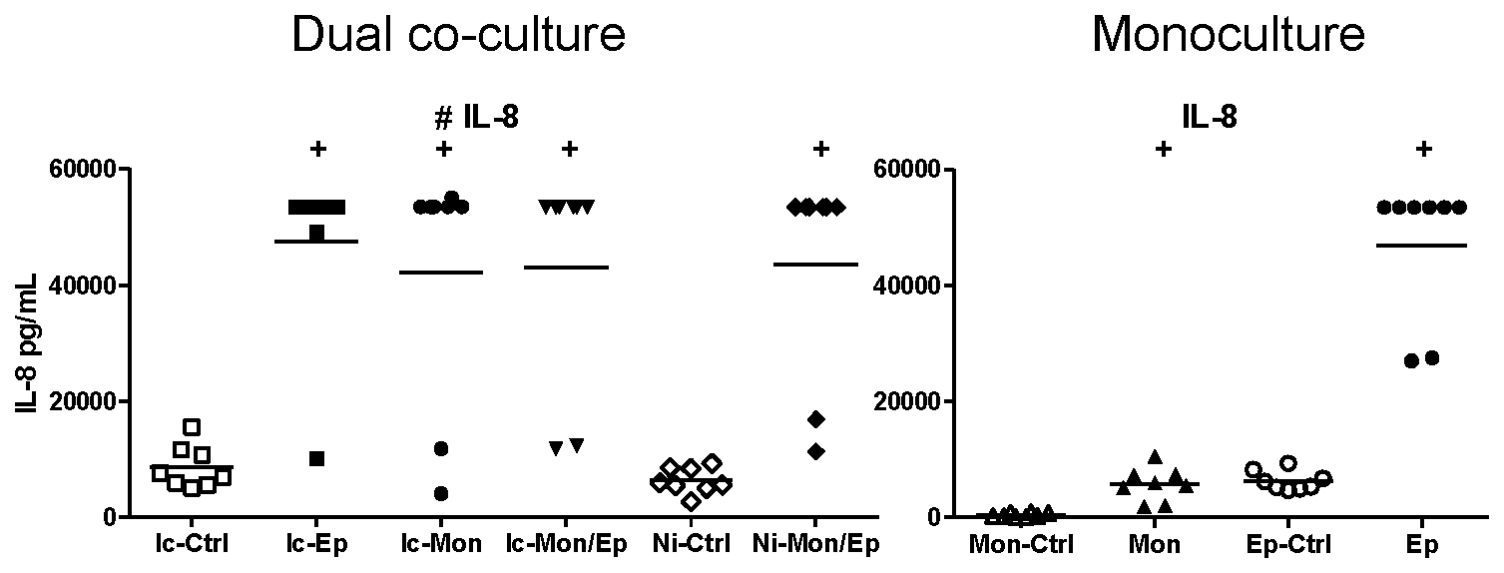
UPEC-induced soluble-dependent biomarker responses in dual co-cultures grouped according to biomarker levels in monocyte monocultures. Experimental Details are as per Figure 4. In this dataset, the least common type of response was a synergistic increase based on a soluble factor(s) conferred by the presence of monocytes. Statistical comparisons are: ^+^ control vs corresponding infected; ^#^ insert-containing infected co-culture vs corresponding infected monoculture; ^┴^ insert-containing infected co-culture vs no insert infected co-culture, notations are p < 0.05; Mann–Whitney U-test.

**Figure 7:**
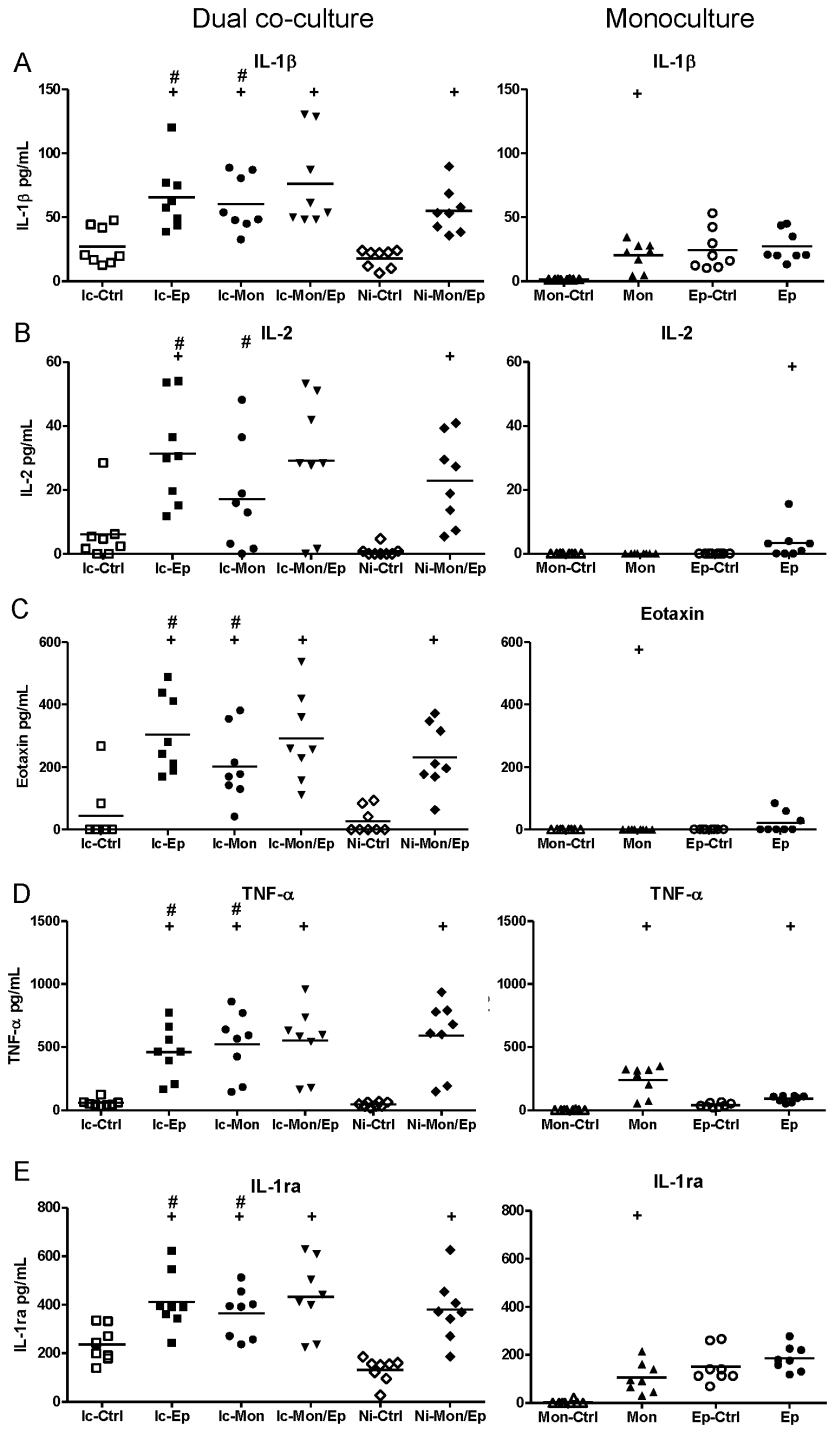
UPEC-induced soluble-dependent biomarker responses in dual co-cultures grouped according to low biomarker levels in monocultures. Experimental Details are as per Figure 4. In this dataset, a common type of response was a synergistic increase based on a soluble factor(s) conferred by the presence of monocytes and uroepithelial cells, with low levels from monocultures absent in dual co-cultures. Statistical comparisons are: ^+^ control vs corresponding infected; ^#^ insert-containing infected co-culture vs corresponding infected monoculture; ^┴^ insert-containing infected co-culture vs no insert infected co-culture, notations are p < 0.05; Mann–Whitney U-test.

BioPlex assays of insert-containing and no-insert dual co-cultures targeting 27 human cytokines, chemokines and growth factors were used to characterize the extent of synergistic interactions within the cultures. These assays yielded numerous interesting response patterns, shown in Figures 4-7. Additional data that are not shown in these Figures are presented as Figures S2-S3 (e.g. biomarker responses in infected monocultures that simply had higher levels than uninfected controls). A summary of significant synergistic and antagonistic interactions in these assays are listed in Table 1 (infected co-cultures), and Table 2 (non-infected co-cultures). 

**Table 1:** Summary of synergistic and antagonistic effects between infected uroepithelial cell and monocyte dual co-cultures compared to monocultures.

UPEC-Infected*			
**Contact**	**Soluble-Ep**	**Soluble-Mon**	**Soluble-both**
IL-10	IL-17	IL-8	IL-1β
IL-6	MCP-1	IL-10	IL-1ra
GM-CSF	MIP-1α		IL-2
PDGF-BB	MIP-1β		Eotaxin
	RANTES		TNF-α
	IP-10		PDGF-BB
	IL-6		
	GM-CSF		

* Biomarker levels with a statistically significant difference (not merely additive effects) compared to biomarker levels in monocultures (Mann-Whitney U-test p < 0.05).

**Table 2:** Summary of synergistic and antagonistic effects between non-infected uroepithelial cell and monocyte dual co-cultures compared to monocultures.

Non-infected*	
**Contact**	**Soluble**
IL-1ra	IL-1ra
IL-2	IL-4
VEGF	IL-9
	IL-10
	FGF basic

* Biomarker levels with a statistically significant difference (not merely additive effects) compared to biomarker levels in monocultures (Mann-Whitney U-test p < 0.05).

The data from these assays showed that biomarker responses occurred differentially as a blend of contact-dependent and soluble factor-mediated responses. The contact-dependent responses are summarized in Figure 4, which illustrates this pattern of response for several biomarkers in addition to IL-10, namely Interleukin-6 (IL-6), Granulocyte Macrophage-Colony Stimulating Factor (GM-CSF), and Platelet-Derived Growth Factor-BB (PDGF-BB). The data for IL-10 also showed that the response as measured by BioPlex followed parallel trends as detected in ELISA using samples from independent assays (Figure 4A): i.e. insert-containing infected dual co-cultures had higher IL-10 levels than monocyte monocultures, and no insert dual co-cultures had significantly higher levels compared to insert-containing dual co-cultures. 

These data also showed that contact-dependent responses to UPEC infection were not always synergistic in nature. For example, the contact-dependent responses noted for IL-6 GM-CSF and PDGF-BB were antagonistic; in contrast to IL-10 (a synergistic contact-dependent response pattern). These factors were produced in significantly lower levels in infected dual co-cultures without inserts compared to insert-containing co-cultures (Figure 4B-D; compare Ni-Mon/Ep [lower] with Ic-Mon/Ep [higher]). Thus, UPEC-triggered production of IL-6, GM-CSF and PDGF-BB in uroepithelial cell-monocyte dual co-cultures is inhibited by contact between the two host cell types. A similar response pattern of contact-dependent antagonism was noted for G-CSF, but this difference did not reach statistical significance (Figure S3). 

In addition to contact-dependent synergism and antagonism, other response patterns for some biomarkers were soluble-dependent. In fact, soluble-dependent interactions were the most common type of responses noted for the biomarkers overall. Among the soluble-dependent responses, we noted three distinct types of increase responses in co-cultures, and we grouped these according to the level of biomarker production in (i) uroepithelial cell monocultures, Figure 5; (ii) monocyte monocultures, Figure 6; and (iii) both monocultures, Figure 7. Synergistic responses in non-infected co-cultures are shown in Figure S2, while biomarkers that had no synergistic responses are presented in Figure S3. We noted that functional classes of biomarkers were not restricted to any one type of response pattern. For example, biomarkers related to recruitment and cell migration, and cell growth/regulation could follow production patterns of (i), (ii) or (iii). Specific examples include biomarkers such as IL-1β, IL-1ra, IL-2, Eotaxin and TNF-α that had patterns of minimal monoculture expression (Figure 7), but were expressed at higher levels in infected dual co-cultures (with or without inserts). Examples where co-cultures had biomarker increases compared to uroepithelial monocultures included IL-17, IP-10, MCP-1, MIP-1α/β and RANTES (compare Ic-Ep and Ep p < 0.05; Figure 5). For other biomarkers, infected uroepithelial monocultures produced the biomarker in the absence monocytes, e.g. TNF-α, IL-4 and IFN-γ (Figure 4B, 4C, S2A, S2F). 

IL-8 had a unique response pattern whereby infected uroepithelial cell monocultures produced high levels, but for monocyte-containing cultures, IL-8 was only produced when in dual co-culture (Figure 6). Other biomarkers, as shown in Figure S2 and S3, provide examples of soluble-dependent interactions that lead to increase based on dual co-cultures without infection. Figure S2 highlights soluble-dependent interactions that caused increased expression when the two cell types were combined in the absence of infection. In many of these cases, the increases due to infection were additive based on monoculture levels. Figure S3 also illustrates purely additive increases of monoculture responses but without interaction effect from non-infected dual co-cultures (as in S2). Thus, Figures S2 and S3 indicate infection-dependent and -independent effects towards biomarker production. As noted, Table 1 summarizes the infection-independent synergistic effects between uroepithelial cells and monocytes, and Table 2 lists the significant interactions in these assays for non-infected co-cultures. Statistical comparisons using ANOVA (Tukey’s post-hoc) showed similar findings of significant differences between groups (except with lower P values); the more conservative results from non-parametric analyses are shown in Tables/Figures. 

## Discussion

Refinement of the cell culture model in the current study from a four cell model to dual co-culture arose with the aim of identifying cell types that add to the IL-10 response to UPEC. We showed that the presence of B-cells conferred a basal level IL-10 response regardless of infection. Monocultures and co-cultures with T-cells showed no increase in IL-10 due to the presence of these cells. These data prompted us to remove both lymphocyte types from the model. Macrophages, representative of cells pre-existing in the bladder [48], were also examined and their inclusion in co-culture with uroepithelial cells promoted a strong increase in the IL-10 response to UPEC. However, the most striking and interesting finding in this study relates to the discovery of a monocyte, but not macrophage-dependent synergistic interaction with bladder uroepithelial cells for UPEC-induced IL-10 production. This raises the role that monocyte extravasation into tissue may play [49], where the cells might differentiate in inflammatory states [50,51]. Their role in cell-cell interactions with uroepithelial cells *in vivo*, which might influence bladder cytokine responses during UTI, is unknown. Mononuclear cell extravasation through epithelia in other models has previously been characterised [52,53], and a robust monocyte inflammatory infiltrate in bladder was recently shown in UPEC UTI [45]. There is minimal data in the literature pertaining to the function and specific localization of infiltrating monocytes in the bladder during UTI and further investigation is required to elucidate this aspect of UTI pathogenesis. The present study is the first to show that uroepithelial cell-monocyte interactions directly modify IL-10 and multiple other cytokine responses triggered by UPEC. These findings have implications for understanding bladder responses to UPEC as well as other uropathogens that induce inflammation in the urinary tract. 

The results of this study confirm that monocytes are the main cell producer of IL-10 in response to UPEC in dual co-culture. Surprisingly, we found that the presence of uroepithelial cells significantly enhances this monocyte IL-10 response to UPEC in a synergistic manner. To our knowledge, there are no prior studies that have reported synergistic contributions of bladder uroepithelial cells to antibacterial responses, highlighting the novelty of these findings. Our qRT-PCR transcriptional analyses showed an increase of IL-10 mRNA synthesis in monocyte co-cultures with uroepithelial cells that were physically separated by inserts. However, uroepithelial cells showed no significant IL-10 transcriptional activity in response to UPEC. In corresponding protein quantitation experiments, we observed a four-fold increase in monocyte-derived IL-10 in dual co-cultures where the cells were separated by inserts, compared to an eight-fold increase in co-cultures were monocytes were not separated from uroepithelial cells. Thus, not only do monocytes contribute the bulk of the IL-10 response to UPEC, which is consistent with a prior study [12], but this response occurs in a synergistic, contact-dependent manner in the presence of uroepithelial cells. Contact-dependent, synergistic interactions have not previously been reported for IL-10 responses during infection. Interestingly, we also noted several biomarkers with response patterns that were mediated by soluble factors and IL-10 was among these also. The significance of these soluble-dependent response patterns is unclear, although it is reasonable to predict that these may contribute to paracrine antimicrobial signalling mechanisms. In our study, we have used the term synergy to describe the IL-10 production effect observed in co-cultures. The literature has indistinct, overlapping definitions of the biological conditions that define synergistic and priming effects. In this sense, the effects of a putative soluble-signal(s) between monocytes and uroepithelial cells could be regarded as a priming strategy. However, the identity of the soluble-dependent mediator(s) of enhanced IL-10 production remains to be determined, and thus synergy provides a more encompassing term than priming, to represent the sum of soluble- and contact-dependent responses analysed within this study.

The synergistic interaction between monocytes and uroepithelial cells for UPEC-triggered IL-10 production discovered in this study may depend, in part, on pathogen ligand signalling through receptors such as TLRs. Promotion and/or antagonism of signalling events for macrophage cytokine synthesis at the axis of multiple TLRs has been described [54,55]. A plethora of priming signals may promote recruitment or cytokine production in mononuclear cells [51,56], and synergistic effects may provide a basis for broader complexity of host responses. Many pathogenesis studies on disease models now use multi-type cellular assays that often lead to more complex views of host responses. In this sense, such models may provide a more realistic view of the cell-cytokine interactions that occur *in situ*, with positive and negative feedback loops based around sequential multifaceted immunological events. The data provided in this study suggest that such feedback loops are relevant to anti-UPEC responses. 

Our BioPlex analysis provides overarching and broad insight into the ample biomarker responses to UPEC beyond IL-10, that are mediated by uroepithelial cell-monocyte interactions. Multiple biomarkers were shown to depend on specific cell arrangements for their peak production in response to UPEC, and we discovered different degrees of contact- and soluble-dependency for biomarker production in a mixture of synergistic and antagonistic interactions. For example, UPEC-induced IL-10 production peaked as a contact-dependent, synergistic response, while IL-6 and GM-CSF production was also contact-dependent but was antagonistic, driven by the presence of the two cell types. PDGF-BB exhibited both soluble-dependent synergy (insert dual co-cultures compared to monocultures), and contact-dependent antagonism (insert versus no-insert dual co-cultures). When grouped according to the contact dependency of the interaction these data reveal that uroepithelial cells can augment biomarker production in response to UPEC when there is a monocyte-derived soluble factor produced. Here, monocytes could trigger uroepithelial cells to produce the cytokine, or a soluble signal(s) from infected uroepithelial cells could prompt monocytes to produce the cytokine, similar to IL-10 but without contact dependency. We note that development of tight-junction uroepithelial monolayers were avoided in the current study to mimic conditions of uroepithelial cell exfoliation in UTI, as reviewed elsewhere [57].

Another cytokine of interest, due to its known role in UPEC UTI, is G-CSF. High levels of G-CSF were detected in uroepithelial cell monocultures and dual co-cultures in this study, but we did not detect a consistent increase in response to infection, which contrasts with the murine model of UPEC UTI [58]. This difference could relate to the human cell-based system that we have used in contrast to the murine model; distinct differences between human and murine macrophages to UPEC in terms of cytokine responses have been reported [47]. Other experimental differences such as time of exposure, UPEC challenge strain, and/or use of fimbriae-enriched bacterial cultures could also explain the lack of infection-induced G-CSF response in the current *in vitro* study compared to the murine UTI model. It is also plausible that cell types excluded from the current work (e.g. neutrophils, dendritic cells, mast cells) may contribute to responses known to occur *in vivo*, which is a limitation of the current study by-design. 

Intimate cooperation between cells of the innate immune system such as epithelial cells, monocytes and neutrophils and their soluble factors is critical for antimicrobial defence. Such cooperative interactions have been demonstrated in several recent *in vitro* and *ex vivo* studies of responses to several pathogens; in *Mycobacterium* spp. infection, soluble factors produced by infected epithelial cells activate neutrophils for diapedesis, and infected neutrophils activate macrophages [59,60]; in *Bordetella pertussis* infection, cell contact rather than production of soluble factors is required for generating suppressive host responses [61]; *Leishmania braziliensis*-infected human monocytes produce soluble factors including IL-10 and C-X-C motif chemokine 10 (CXCL10) (also known as Interferon gamma-induced protein 10; IP-10) that activate and enhance the expression of cytokines in non-infected monocytes [62]; in an *in vitro* co-culture system of *Helicobacter pylori* infection used to model the *in vivo* situation, cooperative interactions between epithelial cells and monocytes related to cathepsin X and cytokine expression are modulated by the *H. pylori* virulence factor CagA [63]; in *Burkholderia pseudomallei* infection, macrophage-lymphocyte cooperative interactions mediate antibacterial activity [64]. Other cooperative cell-cell responses to various pathogens have recently been reviewed [32]. The results of the current study extend our knowledge of these cell-cell interactions in response to UPEC by defining novel cooperative effects of uroepithelial cells and monocytes to drive IL-10 and other biomarker responses. Precisely how the synergistic and antagonistic responses described in this study might influence the pathogenesis of UPEC UTI will require further study and is an area in much need of investigation.

## Supporting Information

Figure S1 **Control cell culture levels with basal level expression of IL-10 not contributing to synergistic effects.** (A) B-cells provide only a higher basal level of IL-10 in co-cultures when present (Non-infected vs. infected co-culture, ^§^ p ≤ 0.001; Presence/absence of B-cells comparing Non-infected, or infected co-cultures, * p ≤ 0.001; SEM bars). (B) Additional non-infected single and co-cultures used in comparison to infected co-cultures from Figure 1A. (C) Non-infected and infected monocultures of uroepithelial cells in comparison to data from Figure 2 (not significant).
(TIF)

Figure S2 **Basal-level biomarker production in non-infected monocultures can contribute to infection-independent synergistic effects in dual co-cultures.** Some dual co-cultures demonstrated a synergistic increase of cytokine production when the two cell types were incubated together, but the only infected condition increase came from additive levels seen in monocultures (Statistical comparisons are: ^+^ control vs corresponding infected; ^#^ insert-containing infected co-culture vs corresponding infected monoculture, notations are p < 0.05; Mann–Whitney U-test).
(TIF)

Figure S3 **Basal-level biomarker production in monocultures can be expressed as additive effects in dual co-cultures.** The remaining cytokines investigated for induction in the dual/monocultures showed a purely additive effect, based on monoculture cytokine production, or no significant increase at all (Statistical comparisons are: ^+^ control vs corresponding infected; ^#^ insert-containing infected co-culture vs corresponding infected monoculture, notations are p < 0.05; Mann–Whitney U-test).
(TIFF)
