# Supplementary figures and images for: Human Bladder Uroepithelial Cells Synergize with Monocytes to Promote IL-10 Synthesis and Other Cytokine Responses to Uropathogenic Escherichia coli
Source: PLoS One. 2013 Oct 14;8(10):e78013. doi: 10.1371/journal.pone.0078013 (PMC3796480; doi:10.1371/journal.pone.0078013)

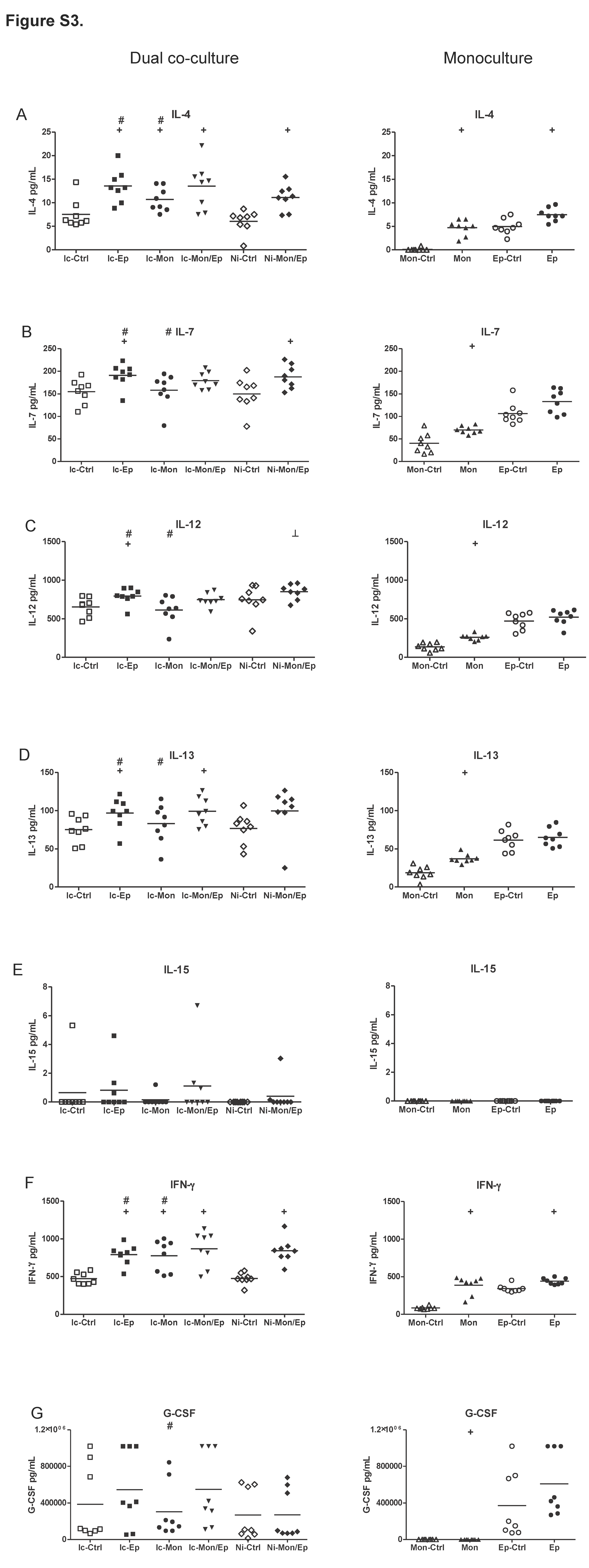

Supplement: Figure S3 — Basal-level biomarker production in monocultures can be expressed as additive effects in dual co-cultures. The remaining cytokines investigated for induction in the dual/monocultures showed a purely additive effect, based on monoculture cytokine production, or no significant increase at all (Statistical comparisons are: + control vs corresponding infected; # insert-containing infected co-culture vs corresponding infected monoculture, notations are p < 0.05; Mann–Whitney U-test). (TIFF) [file pone.0078013.s003.tiff]
